# A combined transcriptomic, epigenetic, and functional analysis identifies novel biomarkers in breast cancer

**DOI:** 10.1186/s41065-025-00630-1

**Published:** 2025-12-27

**Authors:** Mamdooh Gari, Bandar K. Baothman, Khalid Gari, Majid Alhomrani, Haneen Alsehli, Abdullah G. Bagarish, Yasir Hameed, Mohammed Natto, Heba Alkhatabi, Adel Abuzenadah, Sajjad Karim, Jaudah Al-Maghrabi

**Affiliations:** 1 Department of Medical Laboratory Technology, Faculty of Applied Medical Sciences, King Abdulaziz University, Rabigh, Saudi Arabia; 2 Department of Medical Laboratory Sciences, Faculty of Applied Medical Sciences, King Abdulaziz University, Rabigh, Saudi Arabia; 3 Faculty of Medicine in Rabigh, King Abdulaziz University, Rabigh, Saudi Arabia; 4 Department of Clinical Laboratories Sciences, Faculty of Applied Medical Sciences, Taif University, Taif, Saudi Arabia; 5 Research Centre for Health Sciences, Taif University, Taif, Saudi Arabia; 6 Centre for Stem Cell Biology, University of Sheffield, Sheffield, UK; 7 Department of Biochemistry and Biotechnology, The Islamia University of Bahawalpur, Bahawalpur, Pakistan; 8 Department of Medical Laboratory Technology, Faculty of Applied Medical Sciences, King Abdulaziz University, Jeddah, 21589 Saudi Arabia; 9 Department of Medical Laboratory Sciences, Faculty of Applied Medical Sciences, King Abdulaziz University, Jeddah, 21589 Saudi Arabia; 10 Institute of Genomic Medicine Sciences (IGMS), King Abdulaziz University, Jeddah, 21589 Saudi Arabia; 11 Department of Pathology and Laboratory Medicine, Ministry of the National Guard - Health Affairs, Jeddah, 21589 Saudi Arabia

**Keywords:** Breast cancer, Biomarkers, Bioinformatics, Methylation, Immune infiltration, Functional validation

## Abstract

**Background:**

Breast cancer is a leading cause of cancer-related mortality among women worldwide. Identifying reliable molecular biomarkers and therapeutic targets is crucial for improving early diagnosis and treatment strategies. This study aimed to identify and functionally validate key hub genes involved in breast cancer progression using an integrated bioinformatics and experimental approach.

**Methodology:**

Three microarray datasets (GSE42568, GSE29431, and GSE21422) were retrieved from the GEO database to identify differentially expressed genes (DEGs). DEGs common across datasets were subjected to PPI network analysis using STRING and Cytoscape, and hub genes were identified via CytoHubba. The expression of hub genes was validated using RT-qPCR in six breast cancer and five normal epithelial cell lines. Methylation status, survival correlation, immune associations, and drug sensitivity were assessed via GSCA, cBioPortal, OncoDB, and TISIDB. Functional assays, including cell proliferation, colony formation, and wound healing assays were performed following gene overexpression in MCF-7 and T47D cells.

**Results:**

Four hub genes (PPARG, LEP, CD36, and PLIN1) were consistently downregulated in breast cancer and showed higher promoter methylation. Their expression correlated with tumor progression, poor survival, immune infiltration, and drug sensitivity. Functional validation demonstrated that overexpression of each gene reduced proliferation, colony formation, and migration in vitro. Additionally, these genes exhibited subtype-specific immune interactions and drug response profiles, with PPARG emerging as a particularly strong therapeutic biomarker.

**Conclusion:**

This study identified and experimentally validated four hub genes as potential biomarkers and therapeutic targets in breast cancer. Their expression is regulated by methylation and contributes to tumor progression and immune modulation, highlighting their clinical utility in precision oncology.

**Supplementary Information:**

The online version contains supplementary material available at 10.1186/s41065-025-00630-1.

## Introduction

Breast cancer remains one of the most prevalent and deadly malignancies among women worldwide, representing a significant public health burden [1–3]. According to the latest global cancer statistics, breast cancer has surpassed lung cancer as the most commonly diagnosed cancer, with an estimated 2.3 million new cases and approximately 685,000 deaths reported globally in 2024 alone [4, 5]. Despite advances in early detection, surgical techniques, and systemic therapies, including chemotherapy, hormonal therapy, and targeted agents, breast cancer continues to exhibit substantial heterogeneity in its clinical behaviour, treatment response, and patient outcomes [6, 7] This heterogeneity emphasizes the urgent need to identify reliable molecular markers that can improve diagnosis, prognostication, and therapeutic targeting.

Recent technological advancements, particularly in genomics and bioinformatics, have enabled large-scale data integration and analysis, offering unprecedented opportunities to uncover novel genetic signatures associated with cancer pathogenesis [8–10]. In this context, in silico approaches such as gene expression profiling, network analysis, and machine learning models have become invaluable tools for dissecting the complex molecular landscape of breast cancer [11, 12]. These methods allow researchers to identify key driver genes, commonly referred to as “hub genes,” that occupy central positions within regulatory or protein-protein interaction (PPI) networks and are often implicated in critical biological pathways driving tumor development and progression [13–15].

Several studies have reported the identification of hub genes that play pivotal roles in breast cancer. For example, TP53, a well-known tumor suppressor gene, has been frequently identified as a central node in various PPI networks, with its mutations correlating with poor prognosis and resistance to therapy [16–18]. Similarly, genes such as ESR1 (estrogen receptor 1), HER2 (ERBB2), and BRCA1/2 have been extensively studied for their roles in breast cancer pathophysiology and have led to the development of targeted therapies [19, 20]. More recent studies using integrative bioinformatics approaches have highlighted additional hub genes, including CDK1, CCNB1, AURKA, and FOXM1, which are associated with cell cycle regulation and are often overexpressed in aggressive breast cancer subtypes [21–23]. These findings emphasize the dynamic and multifactorial nature of gene regulatory networks in breast cancer and highlight the potential of hub genes as therapeutic and diagnostic targets.

Hub genes have emerged as promising candidates for diagnostic and therapeutic applications, as their dysregulation can significantly impact cancer cell survival, proliferation, invasion, and metastasis [24, 25]. Although PPARG, LEP, CD36, and PLIN1 have been examined individually in certain breast cancer contexts, their collective significance has not been systematically evaluated. Previous studies typically analyze these genes in isolation and do not integrate multi-cohort transcriptomic data, epigenetic profiling, pathway analysis, mutational assessment, and experimental validation within a single framework. A comprehensive investigation that combines these methodological layers is needed to clarify their broader relevance in breast cancer. In this study, we present an integrative assessment of these four genes by combining differential expression analysis, promoter methylation profiling, pathway activity evaluation, survival analysis, and genomic alteration data with in vitro functional assays. The novelty of this work lies in the unified approach that links large-scale bioinformatic findings with direct biological validation, demonstrating the consistent downregulation of PPARG, LEP, CD36, and PLIN1 in breast cancer and confirming their suppressive effects on cell proliferation, clonogenicity, and migration. By synthesizing computational and experimental evidence, this study provides new mechanistic insight into the coordinated involvement of these genes in breast cancer progression and highlights their potential relevance for improving diagnostic and prognostic assessment.

## Methods

### Microarray dataset retrieval and preprocessing

Three publicly available microarray gene expression datasets, GSE42568, GSE29431, and GSE21422, were retrieved from the Gene Expression Omnibus (GEO) database (https://www.ncbi.nlm.nih.gov/geo/) [26]. Each dataset contained gene expression profiles of human breast cancer and normal breast tissue samples. GSE42568 dataset comprised 104 tumour and 17 normal samples; GSE29431 dataset comprised 54 tumour and 12 normal samples; and GSE21422 dataset comprised approximately 14 tumour and 5 normal samples. The raw data were normalized and log2-transformed as required. Probe IDs were annotated using platform-specific annotation files, and probes lacking gene symbols or mapping to multiple genes were excluded. When multiple probes corresponded to the same gene, the probe with the highest average expression was retained. In addition, the GEO dataset GSE45827, which includes 130 breast tumour samples and 11 normal breast tissues, was incorporated specifically for ROC curve analysis due to its larger number of control samples and well-balanced clinical annotation, enabling robust evaluation of the diagnostic performance of hub genes.

### Differential expression and overlapping gene analysis

Differential expression analysis was performed using the limma package in R. Genes with an adjusted *p* value less than 0.05 and an absolute log2 fold change greater than 1 were considered differentially expressed. Volcano plots were generated to visualize the distribution of upregulated and downregulated genes within each dataset. To identify robust gene signatures, Venn diagram analysis was performed using the jvenn web tool [27] to determine overlapping differentially expressed genes across all three datasets.

### PPI network and hub gene selection

The overlapping genes were submitted to the STRING database (version 11.5) [28] to construct a PPI network using a medium confidence score threshold of 0.4. The network was visualized using Cytoscape software (version 3.9.1). Hub genes were identified using the CytoHubba plugin in Cytoscape by applying the Degree centrality algorithm.

### Cell culture and maintenance

Six breast cancer cell lines (MCF7, T47D, MDA MB 231, BT 549, SK BR 3, ZR 75 1) and five non-cancerous breast epithelial cell lines (MCF 10 A, 184A1, HBL 100, HMEC, 184B5) were obtained from ATCC, USA. All cell lines were cultured at 37 °C in a humidified incubator with 5% CO₂. MCF7, T47D, and ZR 75 1 were cultured in RPMI 1640 medium (Thermo Fisher Scientific), while MDA MB 231, BT 549, SK BR 3, and HBL 100 were cultured in DMEM (Thermo Fisher Scientific). All media were supplemented with 10% fetal bovine serum (FBS) (Thermo Fisher Scientific) and 1% penicillin–streptomycin. The non-cancerous cell lines were cultured in Mammary Epithelial Cell Growth Medium (MEGM BulletKit, Lonza, CC 3150) according to ATCC recommendations.

### RNA extraction, cDNA synthesis, and RT qPCR

Total RNA was extracted from all cell lines using TRIzol Reagent (Thermo Fisher Scientific) following the manufacturer’s protocol. RNA concentration and purity were measured using a Nanodrop spectrophotometer. Complementary DNA (cDNA) was synthesized from 1 µg of RNA using the High-Capacity cDNA Reverse Transcription Kit (Thermo Fisher Scientific). RT-qPCR was conducted using TaqMan Gene Expression Assays (Thermo Fisher Scientific) on a QuantStudio 6 Flex Real Time PCR System, with GAPDH as the internal control. The following TaqMan Assay IDs were used: PPARG (Hs01115513_m1), LEP (Hs00174877_m1), CD36 (Hs00354519_m1), PLIN1 (Hs00160173_m1), and GAPDH (Hs02758991_g1). All reactions were performed in triplicate. Relative expression levels were calculated using the 2^^−ΔΔCt^ method.

### Enzyme-linked immunosorbent assay (ELISA)-based protein quantification

Cells were washed with cold PBS and lysed using Pierce™ RIPA Lysis Buffer (Thermo Fisher Scientific) supplemented with Halt™ Protease Inhibitor Cocktail (Cat# 78429). Lysates were clarified by centrifugation at 12,000 × g for 15 min at 4 °C, and total protein concentration was determined using the Pierce™ BCA Protein Assay Kit (Cat# 23227). Quantification of target proteins was performed using the following Thermo Fisher ELISA kits according to manufacturer protocols: PPARG Human ELISA Kit (Cat# OKEH02873), LEP Human ELISA Kit (Cat# KAC2281), CD36 Human ELISA Kit (Cat# ab267614), and PLIN1 Human ELISA Kit (Cat# MBS2019054). In brief, 100 µL of standards and appropriately diluted samples were added to pre-coated wells and incubated for 2 h at room temperature. After washing with Wash Buffer (provided in kits), wells were incubated with HRP-conjugated detection antibodies for 1 h. Following additional washes, TMB Substrate Solution (Cat# N301) was added, and color development was stopped using Stop Solution (2 N sulfuric acid; Thermo Fisher Scientific). Absorbance was measured at 450 nm using a microplate reader, and protein concentrations were calculated from standard curves.

### Transcriptional expression analysis of hub genes

The mRNA expression profiles of the hub genes in breast cancer tissues and normal tissues were analyzed using the GSCA database [29]. The TCGA-BRCA dataset was selected for comparison. Expression data were retrieved in TPM format, and boxplots were generated to visualize differential expression.

### Protein-level validation using immunohistochemistry

Protein expression levels of the hub genes were validated using immunohistochemistry (IHC) images from the HPA database [30]. IHC data for both normal breast tissues and breast cancer tissues were reviewed. Staining intensity and localization (cytoplasmic or nuclear) were assessed semi-quantitatively by comparing available images.

### Gene set enrichment analysis (GSEA)

To explore the biological functions associated with the hub genes, GSEA was performed using the GSCA platform [29].

### DNA methylation analysis

Promoter methylation profiles of the four hub genes were analyzed using the OncoDB database [31], which integrates TCGA DNA methylation data. Beta values representing the percentage of methylation at CpG sites were compared between breast cancer and normal tissue samples across gene regions including promoter, gene body, and exon.

### Correlation between methylation and gene expression

To investigate the relationship between DNA methylation and mRNA expression, Spearman correlation analysis was performed using the GSCA platform [29]. Methylation beta values were correlated with gene expression levels from TCGA-BRCA data.

### Oncogenic pathway activity analysis

The activity of cancer-related pathways associated with hub gene expression was analyzed using the GSCA database. Samples were stratified into high and low expression groups based on median gene expression for each hub gene. Pathway activity scores were calculated for hallmark and canonical signaling pathways.

### Mutation and copy number variation (CNV) profiling and variant classification

Genomic alterations including mutations and CNV in the hub genes were analyzed using the cBioPortal database [32]. The “OncoPrint” and “Mutations” modules were used to visualize gene alterations, including mutations and copy number variations. Mutation types, frequencies, and distribution across patient samples were recorded.

### Survival analysis of hub genes

The prognostic relevance of the hub genes in breast cancer was assessed using the KM Plotter tool [33, 34], which integrates survival data from TCGA, GEO, and EGA databases. The “breast cancer” dataset was selected, and Kaplan–Meier survival curves were generated for overall survival (OS). Patients were stratified into high and low expression groups based on median mRNA expression levels.

### miRNA–mRNA interaction prediction

Putative microRNA regulators of the hub genes were predicted using the TargetScan Human database [35]. Predicted miRNAs targeting hub genes were selected based on high context + + scores and conserved site predictions.

### RT-qPCR validation of MiRNA expression

Total RNA, including small RNAs, was extracted from six breast cancer cell lines and five non-cancerous breast epithelial cell lines using the mirVana™ miRNA Isolation Kit (Thermo Fisher Scientific, AM1560). Complementary DNA was synthesized using the TaqMan™ Advanced miRNA cDNA Synthesis Kit (Thermo Fisher Scientific). Expression levels of the selected miRNAs were measured using TaqMan™ Advanced miRNA Assays (Thermo Fisher Scientific) on a QuantStudio 6 Flex Real Time PCR System. U6 snRNA was used as an internal control. Relative expression was calculated using the 2^^−ΔΔCt^ method. All reactions were performed in triplicate.

### Immune and molecular subtype expression profiling

To assess the immune-related expression patterns of the hub genes in breast cancer, expression data were analyzed across five immune subtypes (C1 to C6) using the TISIDB database [36]. Expression of the hub genes across molecular subtypes of breast cancer “(Basal, Her2, LumA, and LumB)” was also analyzed via the TISIDB platform using TCGA-BRCA data.

### Correlation with immune checkpoint molecules

To explore the relationship between hub genes and immune evasion, correlation analysis was performed between gene expression and known immune-inhibitory markers using TISIDB [36].

### Immune cell infiltration and drug sensitivity analysis

The association between hub gene expression and tumor-infiltrating immune cells was analyzed using the GSCA platform [29]. Data from TCGA-BRCA were used to evaluate correlations between gene expression and 24 immune cell types, including monocytes, neutrophils, macrophages, CD8⁺ T cells, and Tregs. Drug sensitivity correlations were assessed using the GDSC dataset integrated within the GSCA platform [29]. Spearman correlation analysis was used to determine associations between gene expression levels and drug response (IC₅₀ values) for selected compounds.

### Plasmid construction and cell transfection

Full-length coding sequences of PPARG, LEP, CD36, and PLIN1 were cloned into mammalian expression vectors under the control of a CMV promoter. Empty vectors were used as negative controls. Breast cancer cell lines MCF7 and T47D were seeded in 6-well plates at 60 to 70% confluency and transfected with 2 µg of plasmid DNA using Lipofectamine 3000 (Thermo Fisher Scientific, L3000015) following the manufacturer’s protocol. Cells were harvested 48 h post-transfection for downstream assays. Expression after transfection was analyzed using RT-qPCR and Western blot techniques. Western blotting was performed following a previously reported protocol [37, 38], with minor modifications.

### Cell proliferation assay

Cell proliferation was assessed using the Cell Counting Kit-8 (CCK-8) assay (Dojindo, CK04). Transfected MCF7 and T47D cells were seeded in 96-well plates (5 × 10³ cells per well) and incubated for 24, 48, and 72 h. At each time point, 10 µL of CCK-8 solution was added to each well and incubated for 2 h. Absorbance was measured at 450 nm using a microplate reader. Results were normalized to control groups and presented as percentage viability.

### Colony formation assay

For clonogenic assays, 500 transfected cells per well were seeded into 6-well plates and cultured for 10 to 14 days until visible colonies formed. Colonies were fixed with 4% paraformaldehyde and stained with 0.5% crystal violet. Colonies were counted manually under a microscope. Experiments were performed in triplicate.

### Wound healing (scratch) assay

To evaluate cell migration, wound healing assays were performed. Transfected MCF7 and T47D cells were grown to near confluency in 6-well plates, and a linear scratch was made using a sterile pipette tip. Detached cells were removed, and fresh serum-free medium was added. Images were captured at 0 and 24 h using an inverted microscope. Wound closure was quantified using ImageJ software by comparing the wound area at 0 and 24 h.

### Statistical analysis

Data were analyzed using R (v4.2.2) and GraphPad Prism (v9.0). Student’s t test or Wilcoxon test was used for two-group comparisons; ANOVA or Kruskal–Wallis test for multiple groups. Spearman correlation assessed associations. Kaplan–Meier curves with log-rank test evaluated survival. P*-value < 0.05, P**-value < 0.01, and P***-value < 0.001 were considered statistically significant.

## Results

### Dataset retrieval, DEGs exploration, hub genes identification, and in vitro expression analysis

**Fig. 1:**
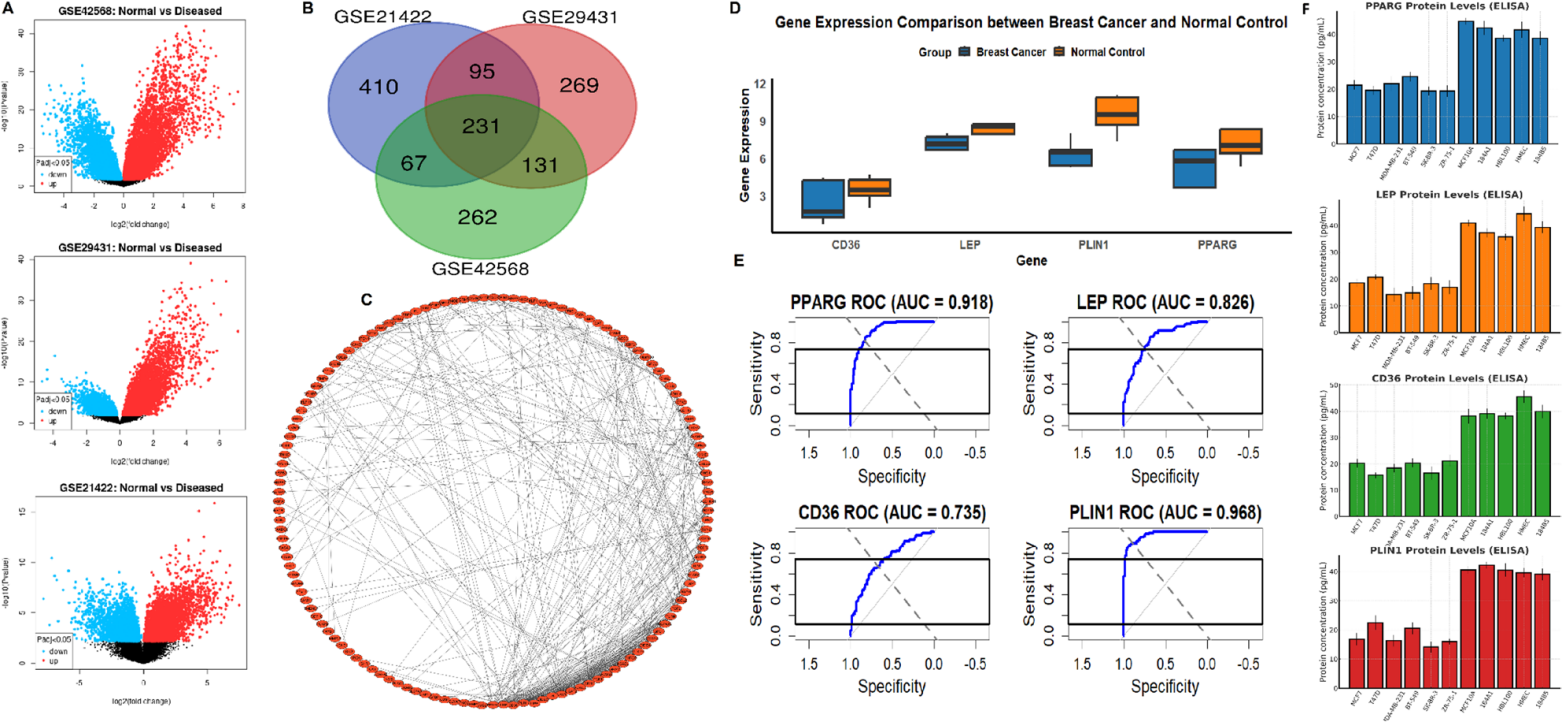
Differential expression analysis and identification of hub genes in breast cancer. **A** Volcano plots showing significantly upregulated (red) and downregulated (blue) genes in three GEO datasets (GSE42568, GSE29431, GSE21422). **B** Venn diagram showing 231 overlapping differentially expressed genes (DEGs) across the three datasets. **C** Protein–protein interaction (PPI) network of overlapping DEGs constructed using STRING and visualized in Cytoscape. **D** RT-qPCR analysis of PPARG, LEP, CD36, and PLIN1 expression in six breast cancer and five normal breast epithelial cell lines. **E** ROC curve analysis of hub genes showing their discriminatory power (AUC) between breast cancer and normal samples. **F** ELISA-based quantification of PPARG, LEP, CD36, and PLIN1 protein levels in the same cell lines, confirming reduced protein expression in cancer cells. *P*-value < 0.05

To explore DEGs, an integrated analysis of three independent microarray datasets (GSE42568, GSE29431, and GSE21422), available in the GEO database, was performed using the limma package. DEGs between breast cancer and normal breast tissues were identified with adjusted p-value < 0.05 and |log2 fold change| > 1. Volcano plots of each dataset revealed distinct sets of significantly upregulated and downregulated genes (Fig. 1A). To enhance the robustness of DEG selection, a Venn diagram analysis was conducted to identify genes commonly dysregulated across all three datasets. This analysis revealed a set of 231 overlapping DEGs (Fig. 1B). Next, PPI network was constructed using the STRING database and visualized in Cytoscape (Fig. 1C). Topological analysis using the CytoHubba plugin and the Degree method identified four hub genes with the highest connectivity: PPARG, LEP, CD36, and PLIN1 (Fig. 1C). The expression patterns of these hub genes were further analyzed using RT-qPCR technique six breast cancer cell lines and five normal breast epithelial cell lines. All four genes were significantly downregulated in breast cancer samples compared to normal controls (Fig. 1D). To evaluate the clinical relevance of the identified hub genes as diagnostic biomarkers, ROC curve analyses were performed using expression data from the GEO dataset GSE45827, which includes a substantial number of tumour and normal breast tissue samples. The AUC values for CD36, LEP, PLIN1, and PPARG all demonstrated strong discriminatory performance between breast cancer and normal tissues (Fig. 1E). Additionally, ELISA-based protein quantification performed across the same panel of breast cancer and normal epithelial cell lines further confirmed that PPARG, LEP, CD36, and PLIN1 proteins were markedly reduced in cancer cells compared with normal controls, consistent with the transcript-level patterns (Fig. 1F).

### Validation of hub gene expression and functional relevance in breast cancer

**Fig. 2:**
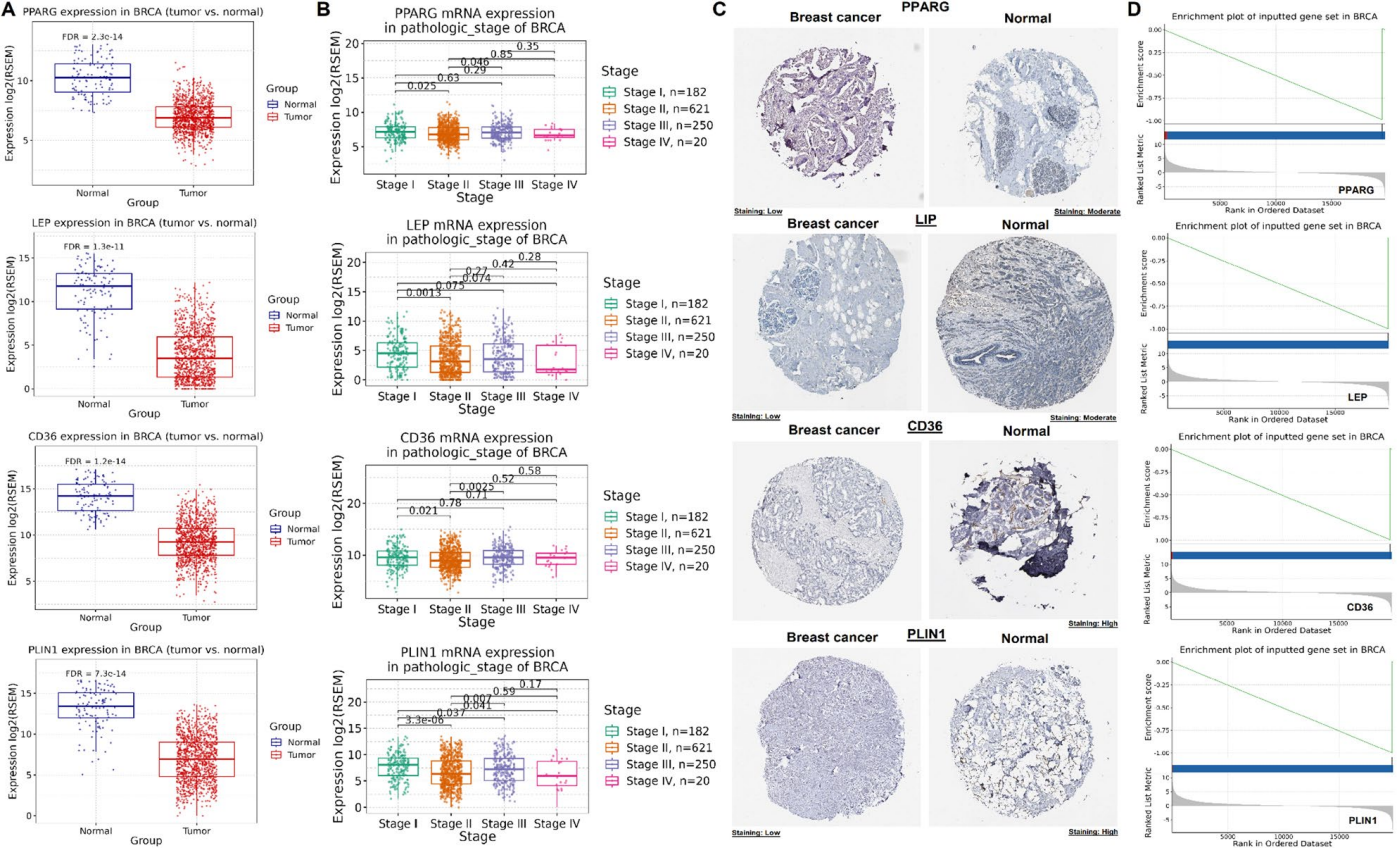
Validation of hub gene expression and clinical relevance in breast cancer. **A** Boxplots showing mRNA expression of hub genes in breast cancer vs. normal tissues using TCGA data (GSCA database). **B** Expression of hub genes across pathological stages I–IV of breast cancer. **C** Representative immunohistochemistry (IHC) images from the HPA showing lower protein expression of hub genes in breast cancer tissues compared to normal. **D** GSEA enrichment plots. P-value < 0.05

To further validate the relevance of the identified hub genes (PPARG, LEP, CD36, and PLIN1) in breast cancer, multiple layers of analysis were conducted using publicly available databases. Firstly, transcriptional expression levels were assessed using GSCA platform. As shown in Fig. 2A, all four hub genes were significantly (p-value < 0.05) downregulated in breast cancer tissues compared to normal tissues. Secondly, to evaluate whether the expression of these genes varies with disease progression, their mRNA levels were analyzed across pathological stages (I–IV) of breast cancer (Fig. 2B). PPARG, LEP, CD36, and PLIN1 all showed a gradual significant (p-value < 0.05) decrease in expression with increasing tumor stage, particularly between stage I and later stages (e.g., stage III/IV), indicating a potential role in disease advancement and aggressiveness (Fig. 2B). Thirdly, to validate protein-level expression, immunohistochemistry (IHC) images from the HPA database were analyzed (Fig. 2C). For all four hub genes, protein expression was lower in breast cancer tissues compared to matched normal tissues (Fig. 2C). Lastly, GSEA was performed using the GSCA database to explore the potential biological relevance of the hub genes in breast cancer (Fig. 2D). All four hub genes exhibited significant negative enrichment scores, suggesting they may be associated with tumor-suppressive pathways that are downregulated in breast cancer (Fig. 2D).

### Methylation status and oncogenic pathway activity analysis of hub genes

**Fig. 3:**
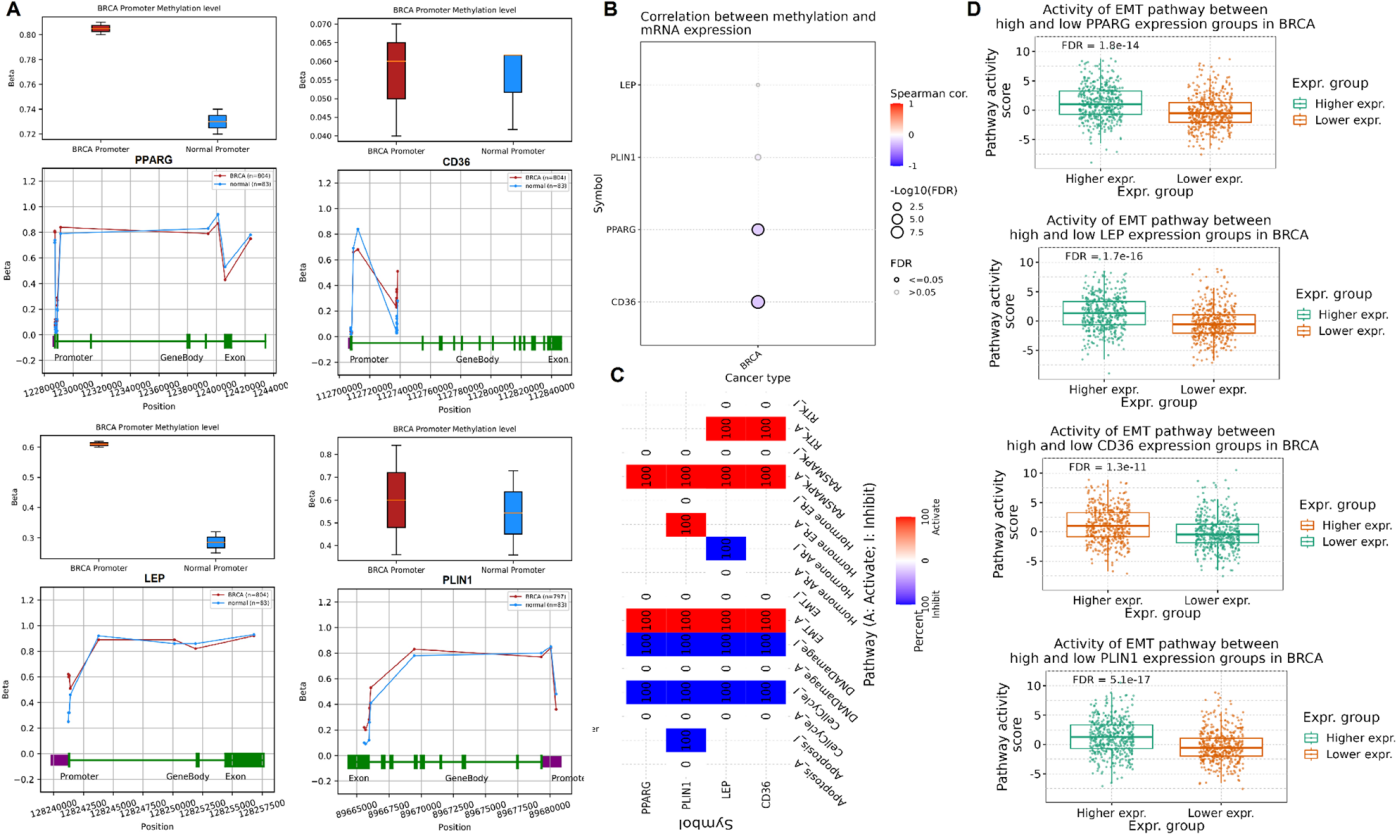
Methylation analysis and oncogenic pathway association of hub genes. **A** Promoter methylation levels and methylation distribution plots across gene regions (promoter, gene body, exon) for each hub gene in breast cancer vs. normal tissues (OncoDB). **B** Spearman correlation between methylation levels and gene expression of hub genes (GSCA). **C** Pathway activity heatmap showing activation or inhibition status of cancer-related pathways for each hub gene. **D** Boxplots showing EMT pathway activity scores between high and low hub gene expression groups in breast cancer samples. *P*-value < 0.05

To explore the potential epigenetic regulation of the identified hub genes, promoter methylation analysis was conducted using the OncoDB database. As shown in Fig. 3A, all four genes exhibited significantly (*p*-value < 0.05) higher promoter methylation levels in breast cancer tissues compared to normal tissues (Fig. 3A). This hypermethylation was consistent across gene regions (promoter, gene body, and exon) Fig. 3A, suggesting a role for DNA methylation in transcriptional repression of these genes in breast cancer. To further examine the impact of DNA methylation on gene expression, Spearman correlation analysis between methylation levels and mRNA expression was performed using the GSCA database (Fig. 3B). PPARG, LEP, CD36, and PLIN1 showed negative correlations (FDR < 0.05), indicating that promoter hypermethylation is likely contributing to their downregulation in breast cancer (Fig. 3B). Next, oncogenic pathway activity analysis was performed to assess whether the hub genes were associated with activation or inhibition of key cancer-related pathways (Fig. 3C). The analysis revealed that PPARG, LEP, and PLIN1 were primarily associated with inhibition of pathways involved in epithelial–mesenchymal transition (EMT), DNA damage response, apoptosis, and hormone signaling. In contrast, CD36 exhibited variable associations, including potential activation of pathways like EMT and glycolysis (Fig. 3C). To validate the relationship between gene expression and pathway activity, the EMT pathway was selected as a representative example, given its known role in cancer invasion and metastasis. As shown in Fig. 3D, lower expression of each hub gene (PPARG, LEP, CD36, and PLIN1) was significantly (p-value < 0.05) associated with increased EMT pathway activity in breast cancer samples (FDR < 1e − 11 for all genes).

### Genomic alteration analysis of hub genes

**Fig. 4:**
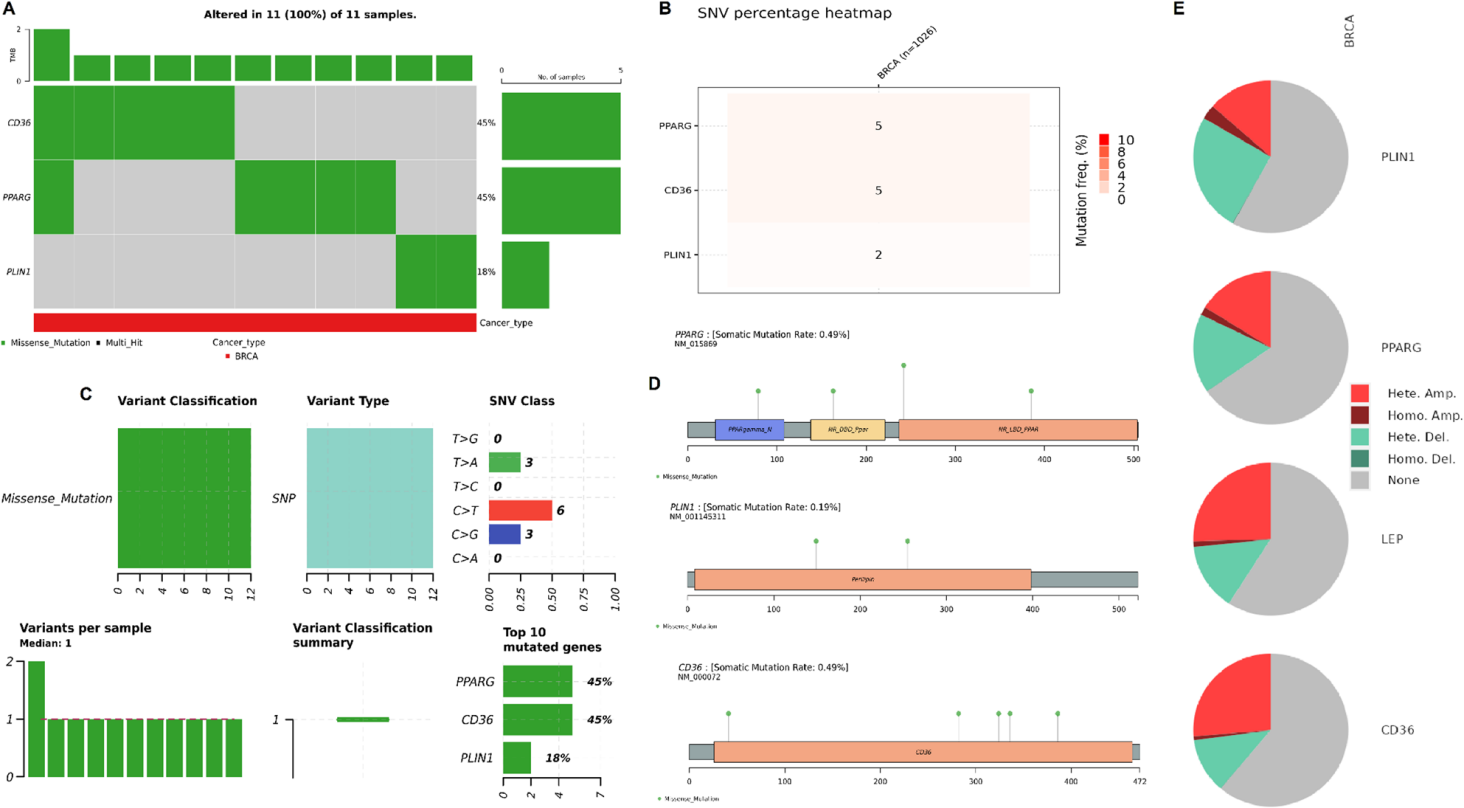
Genomic alteration profiles of hub genes in breast cancer. **A** Oncoprint showing mutation frequency and types of hub genes in breast cancer samples (cBioPortal). **B** Heatmap of single nucleotide variant (SNV) percentages across hub genes. **C** Classification of mutation types, SNV classes, and mutation frequencies in hub genes. **D** Lollipop plots showing mutation positions across protein domains of PPARG, CD36, and PLIN1. **E** Pie charts illustrating copy number alterations (CNA) including amplifications and deletions for each hub gene. *P*-value < 0.05

To investigate the genomic landscape of the identified hub genes in breast cancer, genomic alteration analysis was conducted using the cBioPortal database. As shown in Fig. 4A-B, mutations were observed in all 11 breast cancer samples analyzed, with CD36 and PPARG each altered in 45% of samples, and PLIN1 in 18%, while no mutation was found in LEP genes (Fig. 4A-B). All observed variants were single nucleotide polymorphisms (SNPs), with the majority classified as T > A and C > G transitions (Fig. 4C). All mutations were classified as missense mutations, suggesting possible effects on protein structure and function (Fig. 4C). Lollipop plots in Fig. 4D illustrate the distribution of mutations across functional domains of each protein. Mutations in PPARG occurred in both the DNA-binding and ligand-binding domains, while CD36 and PLIN1 mutations were spread across their respective functional regions (Fig. 4D). Finally, CNV analysis shown in Fig. 4E revealed a mix of heterozygous and homozygous amplifications and deletions. PPARG and CD36 displayed notable frequencies of both heterozygous amplifications and deletions, while LEP and PLIN1 showed fewer alterations (Fig. 4E).

### miRNA-mediated regulation and prognostic significance of hub genes

**Fig. 5:**
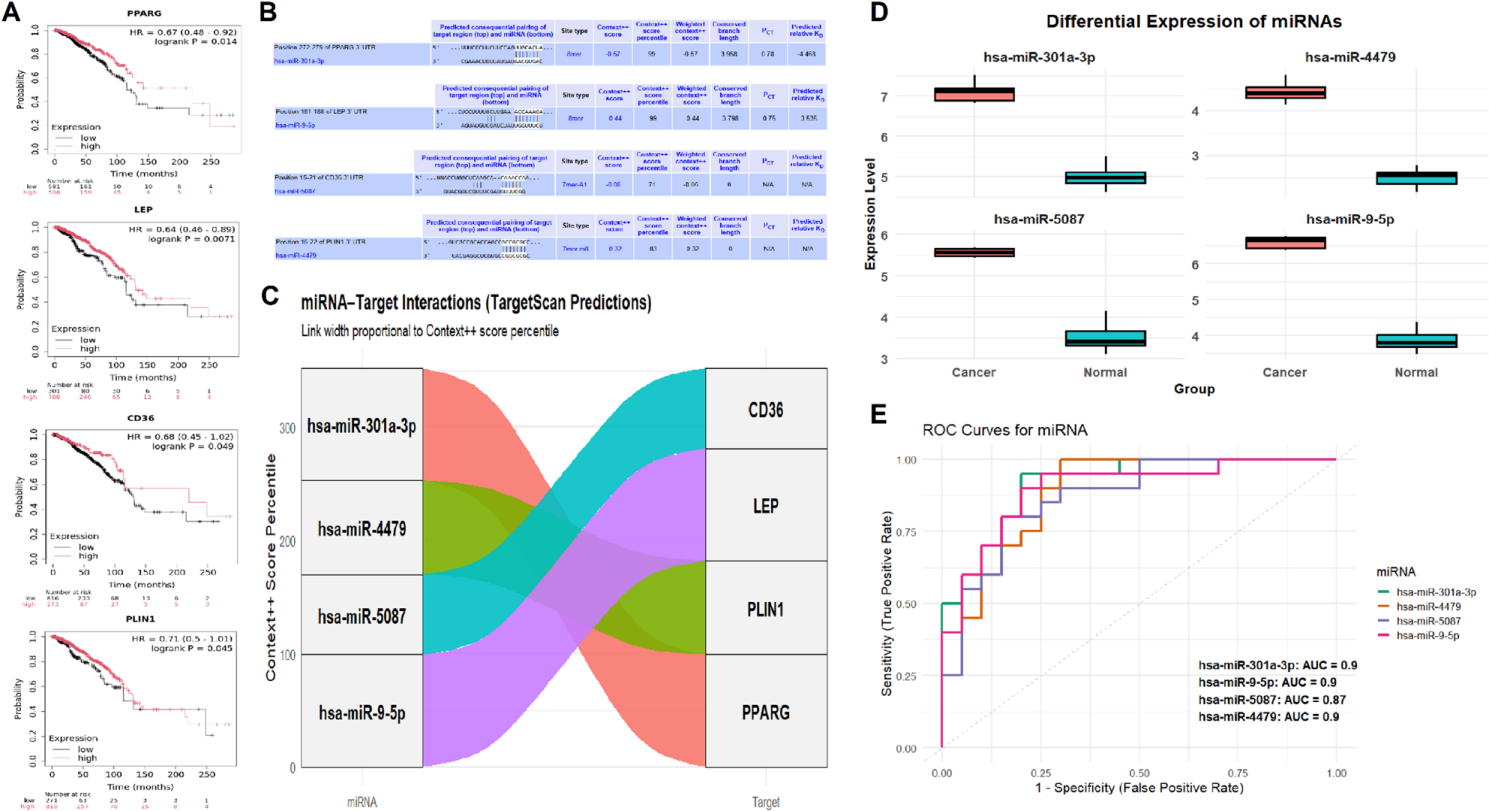
miRNA-mediated regulation and prognostic relevance of hub genes. **A** Kaplan–Meier survival curves showing overall survival differences between high and low expression groups of hub genes. **B** TargetScan-predicted binding site information for miRNAs targeting hub genes. **C** Sankey diagram illustrating predicted miRNA–hub gene interactions with context + + scores. **D** RT-qPCR analysis showing elevated expression of four regulatory miRNAs in breast cancer vs. normal breast epithelial cells. **E** ROC curves of selected miRNAs (miR-301a-3p, miR-4479, miR-5087, and miR-9-5p) showing diagnostic performance. *P*-value < 0.05

To evaluate the clinical relevance of the identified hub genes and their potential post-transcriptional regulation, a comprehensive analysis of prognostic outcomes and miRNA interactions was conducted. Kaplan–Meier survival analysis was performed using the KM Plotter tool to assess the prognostic impact of gene expression levels (Fig. 5A). Low expression of PPARG, LEP, CD36, and PLIN1 was significantly (p-value < 0.05) associated with poor overall survival in breast cancer patients (Fig. 5A). To explore potential regulatory mechanisms, miRNA–mRNA interaction predictions were performed using the TargetScan database (Fig. 5B and C). Four miRNAs were predicted to target these hub genes, including hsa-miR-301a-3p, hsa-miR-4479, hsa-miR-5087, and hsa-miR-9-5p (Fig. 5B and C). Expression levels of the predicted miRNAs were then validated using RT-qPCR across six breast cancer cell lines and five normal controls (Fig. 5D). All four miRNAs were significantly (p-value < 0.05) upregulated in breast cancer cells compared to normal cells. To assess the diagnostic potential of these miRNAs, ROC curve analysis was performed (Fig. 5E). All four miRNAs demonstrated strong discriminatory power for breast cancer detection, with AUC values ranging from 0.87 to 0.90, indicating high sensitivity and specificity (Fig. 5E).

### Expression analysis across molecular subtypes, immune subtypes and performing drug sensitivity analysis of hub genes

**Fig. 6:**
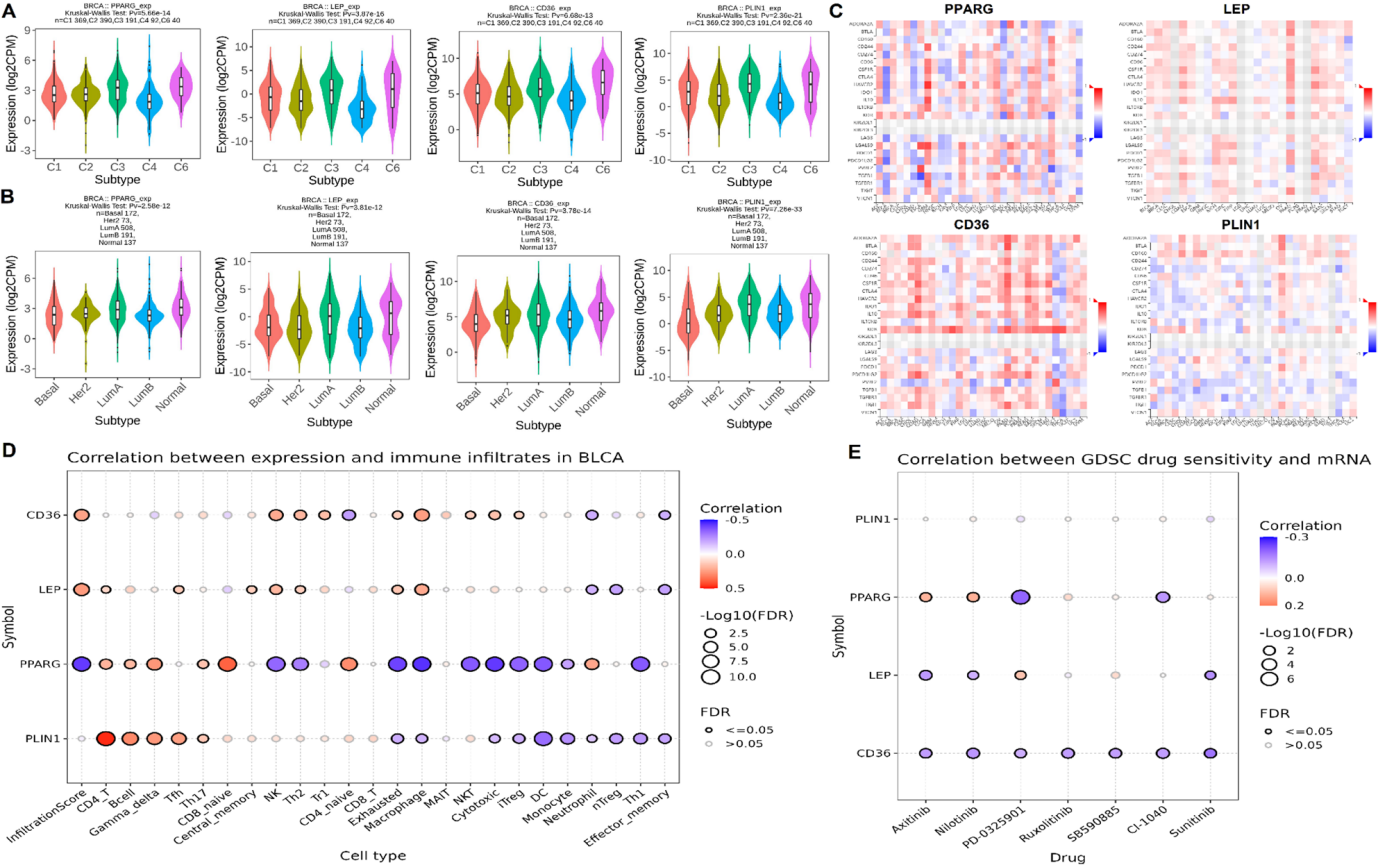
Immunological relevance and drug sensitivity of hub genes in breast cancer. **A** Violin plots showing expression levels of hub genes across six immune subtypes (C1–C6) of breast cancer (TISIDB). **B** Violin plots showing expression levels of hub genes across molecular subtypes (Basal, Her2, LumA, LumB) and normal samples. **C** Heatmaps showing correlation between hub genes and immune checkpoint molecules. **D** Dot plots showing correlation between hub genes and various immune cell infiltrates in breast cancer (GSCA). **E** Drug sensitivity correlations of hub genes with targeted therapies based on GDSC database analysis. P-value < 0.05

To investigate the immunological roles and therapeutic relevance of the identified hub genes in breast cancer, a comprehensive multi-omic analysis was conducted using the TISIDB and GSCA platforms. First, gene expression was analyzed across five immune subtypes of breast cancer (C1–C6, excluding C5) using the TISIDB database (Fig. 6A). All four hub genes exhibited significantly variable expression across immune subtypes (*p* < 0.001), suggesting subtype-specific immunoregulatory functions (Fig. 6A). Notably, PPARG and LEP showed lower expression in immune-suppressed subtypes (e.g., C4), while CD36 and PLIN1 displayed broader heterogeneity (Fig. 6A). Second, the expression of hub genes was evaluated across molecular subtypes of breast cancer, including Basal-like, Her2-enriched, Luminal A, Luminal B, and Normal-like. All four genes again displayed significant subtype-specific differences (Kruskal–Wallis *p* < 1 × 10⁻¹²). Notably, Luminal A tumors showed consistently higher expression of PPARG, LEP, CD36, and PLIN1 compared with and Luminal B, Basal-like, and Her2-enriched tumors (Fig. 6B). Moreover, Normal-like tissues exhibited lower expression levels of PPARG, LEP, CD36, and PLIN1 across all the molecular subtypes of breast cancer (Fig. 6B). These findings align with the known metabolic characteristics of luminal breast cancers, which retain features of differentiated epithelial biology and exhibit higher activity of adipogenic and lipid-handling pathways relative to basal-like tumors. Third, the correlation between hub gene expression and immune inhibitor-related molecules was assessed using the TISIDB database (Fig. 6C). Heatmaps revealed significant correlations between hub genes and several immune checkpoint molecules. For instance, PPARG and LEP negatively correlated with immune-inhibitory markers such as PVRL-2 and KIR2DL3 in breast cancer (Fig. 6C), suggesting their potential involvement in modulating immune evasion in breast cancer. Fourth, to assess immune cell infiltration, GSCA analysis revealed that hub genes were significantly associated with various immune cell populations in breast cancer (Fig. 6D). PPARG, LEP, and PLIN1 showed negative correlations with monocytes cells, neutrophils cells, and effector_memory cells (Fig. 6D). In contrast, CD36 demonstrated a positive correlation with multiple immune infiltrates, including macrophages and Tregs (Fig. 6D). Finally, drug sensitivity analysis was conducted using the GDSC database via GSCA (Fig. 6E). Among the hub genes, PPARG showed a significant correlation with sensitivity to multiple targeted therapies, including PD-0325901, Ruxolitinib, and Nilotinib, indicating that its expression may influence treatment response. CD36, LEP, and PLIN1 showed weaker or no significant drug associations, highlighting PPARG as a promising predictive biomarker for therapeutic stratification (Fig. 6E).

### Functional validation of hub genes by overexpression in breast cancer cell lines

**Fig. 7:**
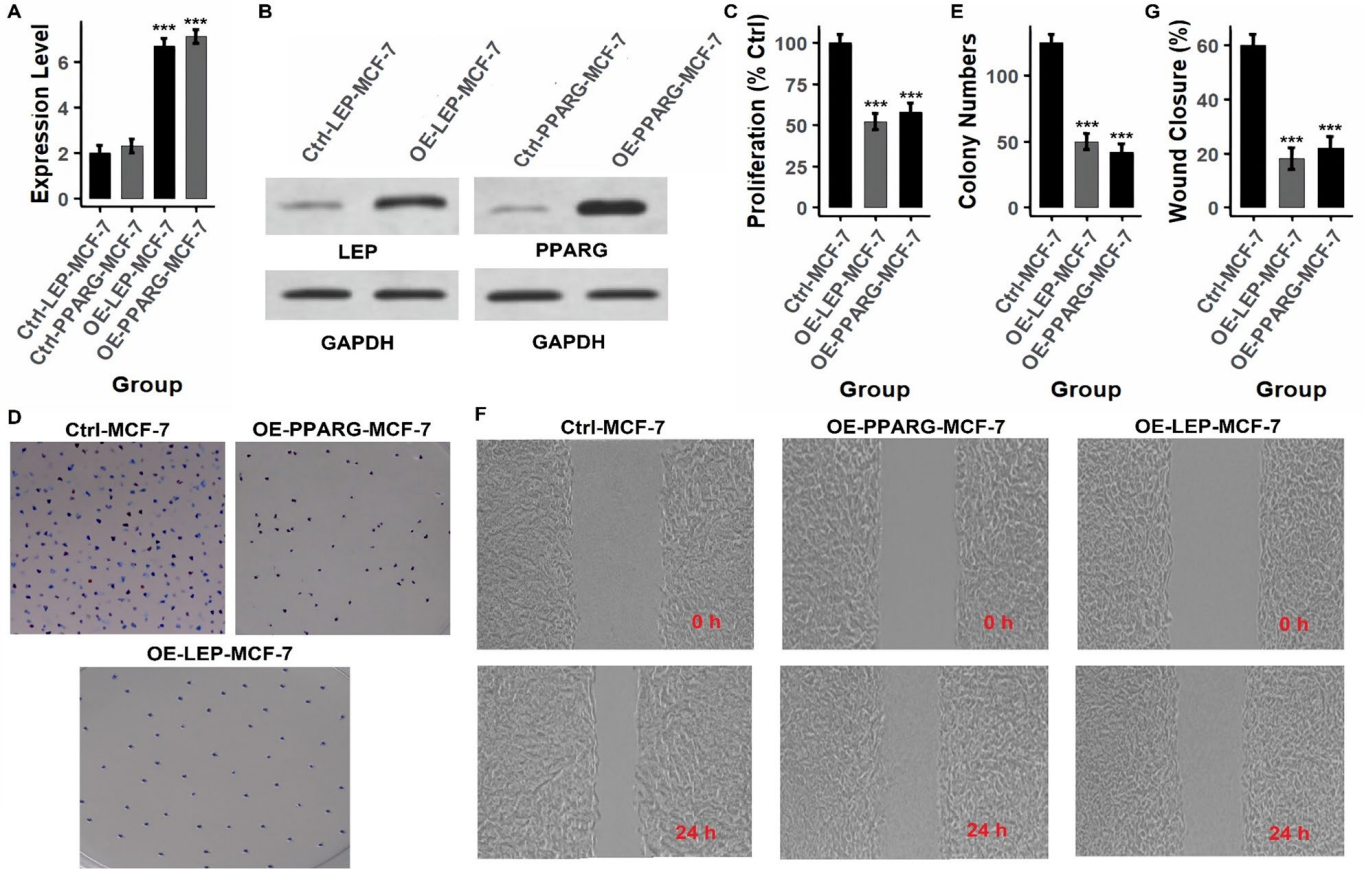
Overexpression of PPARG and LEP suppresses proliferation and migration in MCF-7 cells. **A**,** B** RT-qPCR and Western blot confirm successful overexpression of PPARG and LEP in MCF-7 cells. **C** Proliferation assays show significant reduction in cell growth in overexpression groups compared to controls. **D**, **E** Colony formation assays reveal fewer and smaller colonies following PPARG and LEP overexpression. **F**, **G** Wound healing assays demonstrate impaired migration in overexpressing cells at 24 h. P***-value < 0.001

**Fig. 8:**
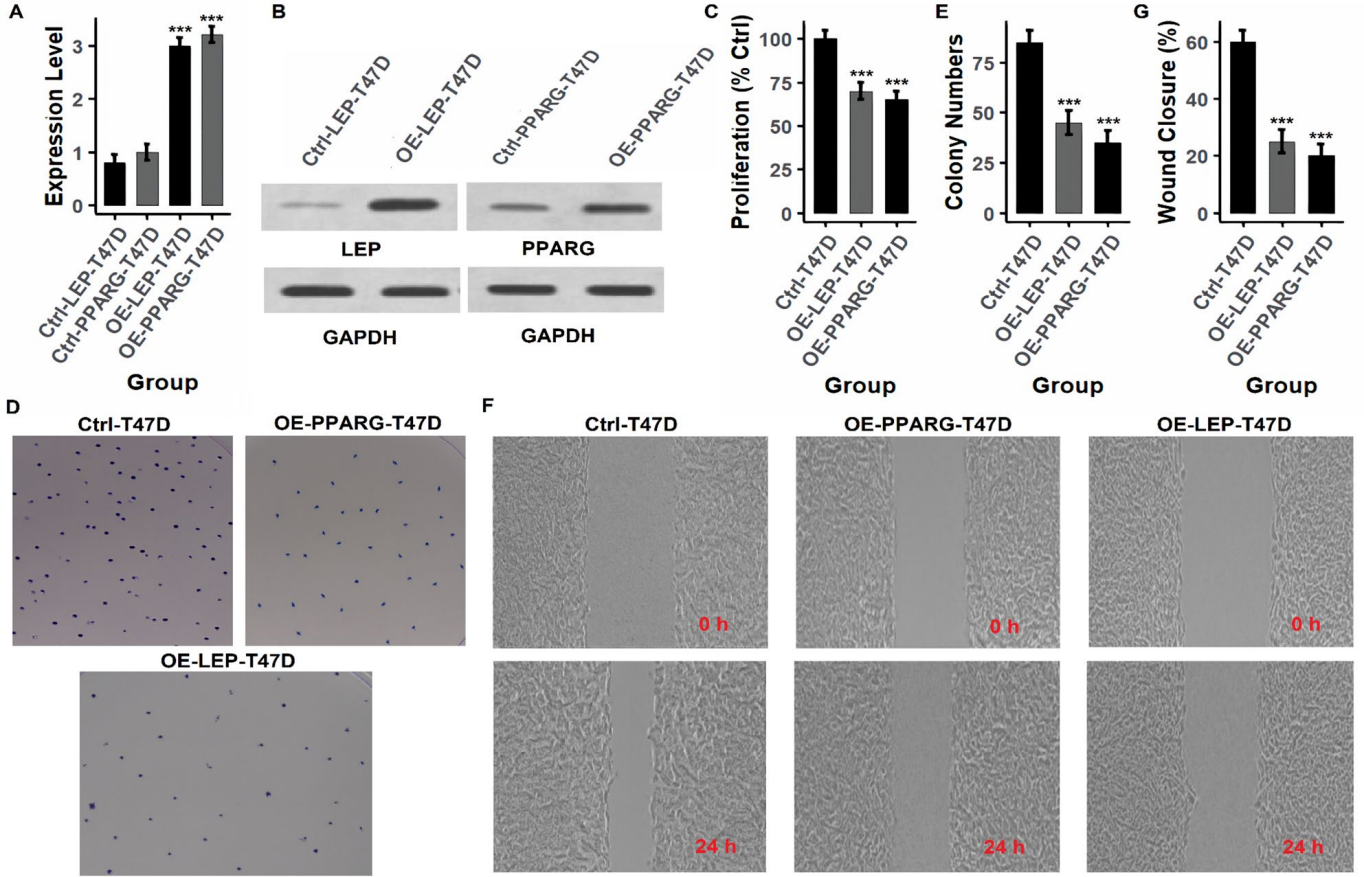
PPARG and LEP overexpression limits tumorigenic properties in T47D cells. **A**, **B** RT-qPCR and Western blot show elevated expression of PPARG and LEP post-transfection. **C** Proliferation is significantly reduced in overexpressing cells. **D**, **E** Colony formation is suppressed. **F**, **G** Wound healing assays indicate decreased migratory ability. P***-value < 0.001

**Fig. 9:**
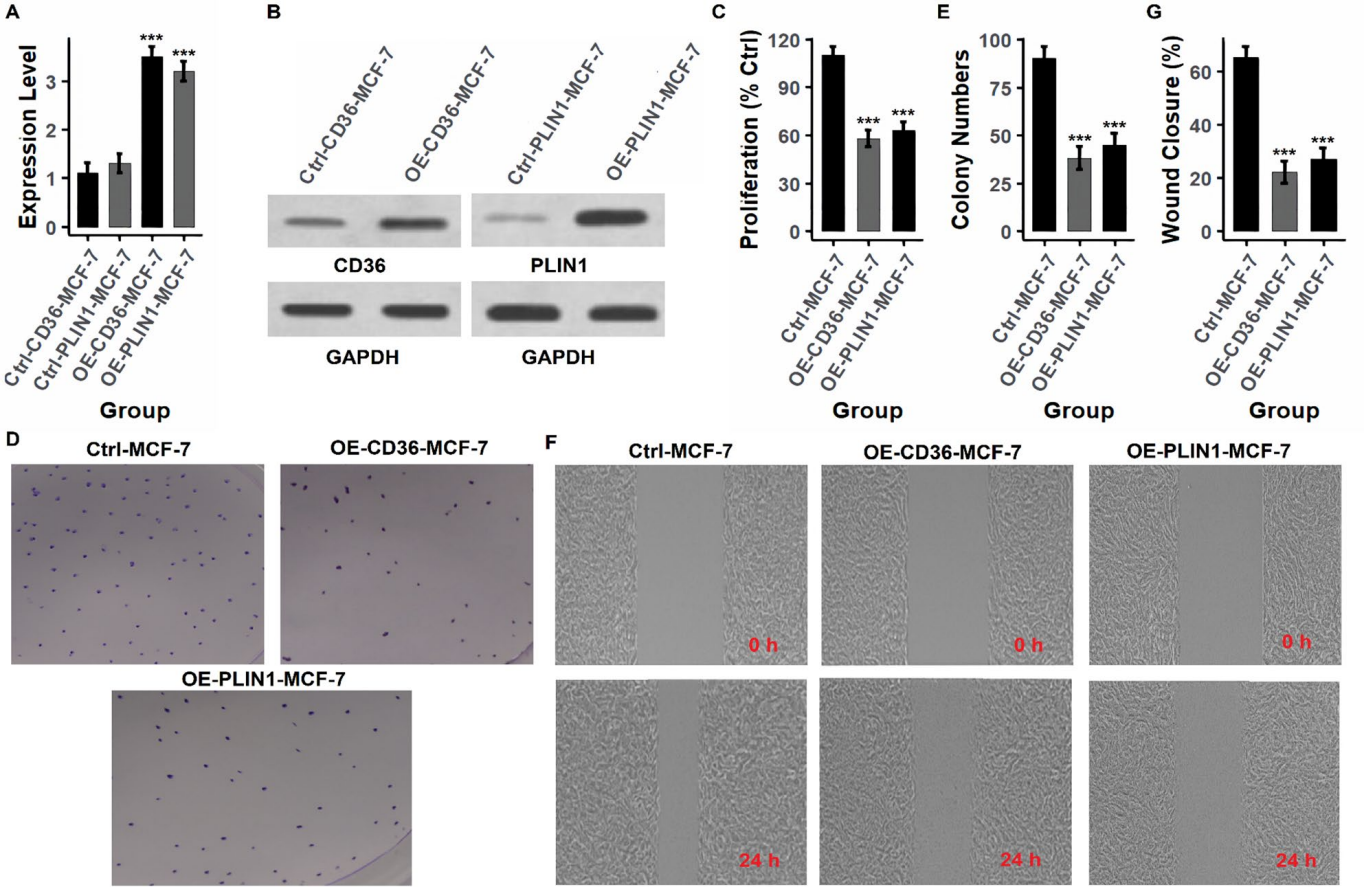
Overexpression of CD36 and PLIN1 inhibits oncogenic behavior in MCF-7 cells. **A**, **B** mRNA and protein levels of CD36 and PLIN1 are significantly increased in MCF-7 cells transfected with expression vectors. **C** Overexpression significantly reduces proliferation (*p* < 0.001). **D**, **E** Colony formation is markedly suppressed. **F**, **G** Wound closure is reduced, indicating decreased cell motility. P***-value < 0.001

**Fig. 10:**
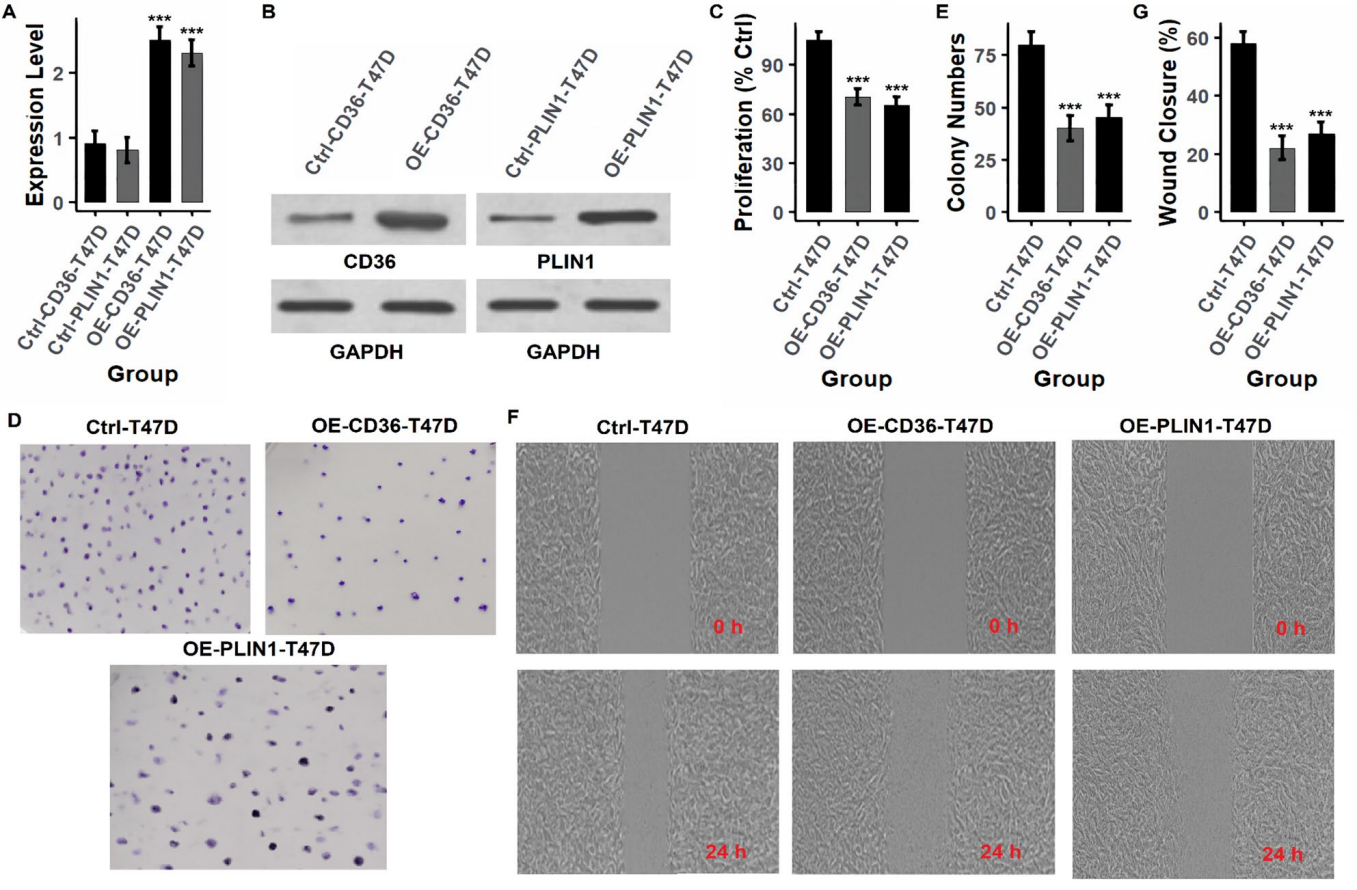
CD36 and PLIN1 overexpression reduces proliferation and migration in T47D cells. **A**, **B** Confirmation of CD36 and PLIN1 overexpression at transcript and protein levels. **C** Cell proliferation is significantly inhibited in both overexpression groups. **D**, **E** Fewer colonies are formed, indicating reduced clonogenic capacity. **F**, **G** Migration is impaired in wound healing assays at 24 h. P***-value < 0.001

To investigate the tumor-suppressive roles of the identified hub genes (PPARG, LEP, CD36, and PLIN1), functional assays were conducted following gene overexpression in breast cancer cell lines (MCF-7 and T47D) using expression vectors. Gene overexpression was first confirmed at both mRNA and protein levels. As shown in Figs. 7A and B and 8A and B (MCF-7) and Figs. 9A and B and 10A and B (T47D), and supplementary data Fig. 1, overexpression of each gene (OE-PPARG, OE-LEP, OE-CD36, OE-PLIN1) resulted in significantly (p-value < 0.001) elevated transcript and protein levels compared to their respective controls. Proliferation assays (Figs. 7C, 8C and 9C, and 10C) demonstrated that overexpression of each hub gene significantly reduced cell proliferation in both MCF-7 and T47D cell lines (*p* < 0.001), indicating growth-inhibitory effects. Consistently, colony formation assays (Figs. 7D-E, 8D-E and 9D-E, and 10D-E) revealed a substantial decrease in colony number upon overexpression of each gene, further confirming their anti-proliferative potential. Similarly, wound healing assays (Figs. 7F–G, 8F–G and 9F–G, and 10F-G) showed lower wound closure in all overexpression groups at 24 h, suggesting that these genes suppress cellular motility and migration.

**Fig. 11:**
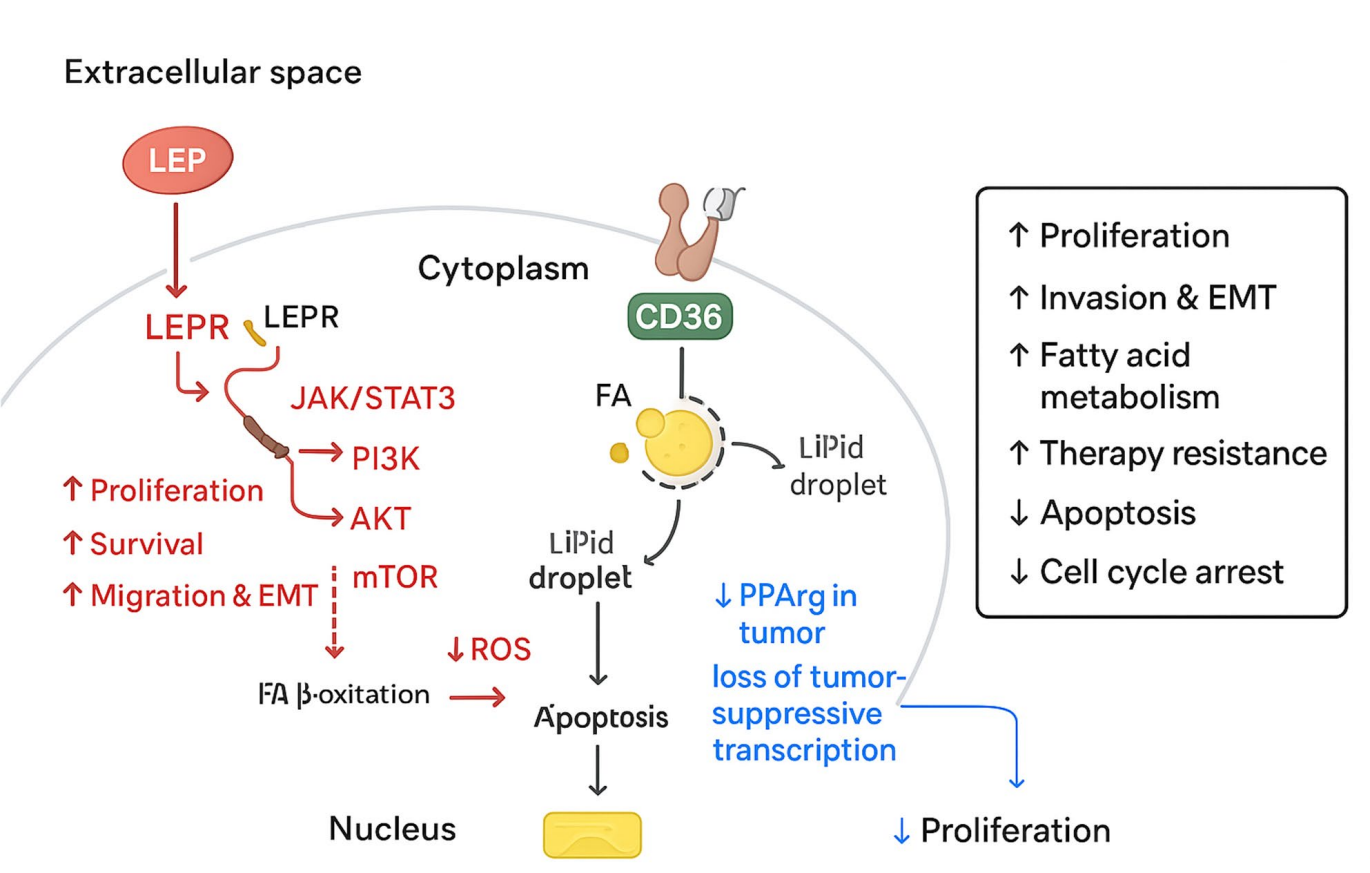
Mechanistic model of hub gene functions in breast cancer progression. Schematic pathway illustrating the regulatory roles of LEP, CD36, and PPARG in breast cancer

Lastly, to elucidate the potential mechanistic roles of the identified hub genes (LEP, CD36, and PPARG) in breast cancer, a comprehensive pathway model was constructed based on integrative molecular and functional analyses. LEP (leptin), secreted into the extracellular space, binds to its receptor LEPR, activating downstream JAK/STAT3 and PI3K/AKT/mTOR signaling pathways (Fig. 11). This cascade promotes cell proliferation, survival, migration, and EMT), key processes involved in tumor progression (Fig. 11). Additionally, mTOR-mediated suppression of fatty acid β-oxidation and reduction of reactive oxygen species (ROS) further supports cell survival and therapy resistance (Fig. 11). CD36, a fatty acid transporter located at the plasma membrane, facilitates fatty acid (FA) uptake and accumulation into lipid droplets, which can protect cancer cells from lipotoxicity and apoptosis. This mechanism contributes to enhanced fatty acid metabolism, EMT, and therapy resistance (Fig. 11). In contrast, PPARG acts as a nuclear tumor suppressor, regulating lipid metabolism and anti-proliferative transcription (Fig. 11). However, in breast cancer, PPARG expression is significantly reduced, leading to a loss of tumor-suppressive transcriptional programs (Fig. 11). This downregulation results in unchecked proliferation and metabolic reprogramming (Fig. 11).

## Discussion

In this study, we employed a comprehensive multi-omics and experimental approach to identify, validate, and characterize four key hub genes (PPARG, LEP, CD36, and PLIN1) as potential biomarkers in breast cancer [39–41]. Through rigorous bioinformatics integration of three independent GEO datasets and validation in cellular models, we revealed their relevance in disease progression, regulation, and potential therapy response. The identification of 231 consistently dysregulated genes across three GEO datasets reinforces the robustness of our analysis pipeline. Several recent studies have demonstrated the importance of using integrated datasets to overcome single-cohort variability and enhance biomarker reliability [42–44]. Within this intersection, PPARG, LEP, CD36, and PLIN1 emerged as top-ranking hub genes in the PPI network, based on degree centrality analysis using CytoHubba. While each gene has been previously implicated in cancer biology, our study uniquely positions them as co-expressed, co-regulated candidate biomarkers in breast cancer.

Our transcriptional analysis using RT-qPCR, TCGA, and GSCA databases confirmed that all four hub genes were significantly downregulated in breast cancer tissues compared to normal controls. Previous studies have linked PPARG downregulation with poor outcomes in various malignancies, including breast and colorectal cancers [45–47]. Similarly, LEP and LEPR signaling has been reported to influence breast cancer development via JAK/STAT3 and PI3K/AKT signaling pathways [48–51], though evidence remains mixed. CD36, classically known as a fatty acid translocase, has been studied in breast cancer metastasis, particularly in stem-like cells [52, 53], while PLIN1, a lipid droplet-associated protein, has been minimally explored in breast cancer, making our findings on its consistent downregulation particularly novel.

Moreover, we found that the expression of these hub genes significantly decreased with advancing breast cancer stage, suggesting their potential value in early detection and prognostic stratification. While some prior work has demonstrated stage-specific expression patterns for PPARG [54], comprehensive stage-wise trends across these four genes have not been systematically reported, emphasizing the novelty of this aspect of our study. We further explored the epigenetic regulation of these biomarkers. Promoter hypermethylation was observed for all four genes in breast cancer tissues. The negative correlation between DNA methylation and gene expression is in line with previous findings for PPARG in lung and colorectal cancers [55, 56]. However, PLIN1 and LEP methylation in breast cancer has been underreported, and our study provides the first integrated view of their epigenetic regulation in this context. Our mutation analysis indicated a low-to-moderate frequency of missense mutations in PPARG, CD36, and PLIN1, with mutation hotspots localized to functional domains. This supports prior findings of PPARG mutations in breast and bladder cancers [57, 58], but adds new knowledge regarding the genomic alteration landscape of PLIN1 and CD36, which remains sparsely reported in breast cancer.

Post-transcriptional regulation was also examined. We identified hsa-miR-301a-3p, hsa-miR-4479, hsa-miR-5087, and hsa-miR-9-5p as potential miRNA regulators of the hub genes. Prior studies have shown that miR-301a promotes breast cancer progression [59] and miR-9-5p is linked to metastasis and EMT [60]. However, our demonstration of their direct regulatory connection to PLIN1 and LEP adds novel insights into the post-transcriptional landscape in breast cancer.

In the context of immune regulation, our findings show that PPARG, LEP, and PLIN1 negatively correlate with various immune cell types, while CD36 is positively associated with Tregs and macrophages. These observations are consistent with prior studies linking PPARG to immune cell polarization [61] and CD36 to immune-suppressive niches [50, 62]. Drug sensitivity analysis revealed that PPARG expression correlates negatively with response to inhibitors such as PD-0325901 (MEK inhibitor) and Ruxolitinib (JAK inhibitor). This aligns with studies that link PPARG activation to enhanced drug sensitivity in breast and gastric cancers [63, 64]. The lack of such correlations for LEP, CD36, and PLIN1 emphasizes the specific predictive potential of PPARG as a pharmacogenomic biomarker in breast cancer.

Our in vitro experiments further validated the functional relevance of these biomarkers. Overexpression of each gene in MCF-7 and T47D cell lines significantly inhibited cell proliferation, migration, and colony formation. While previous studies have demonstrated anti-proliferative effects of PPARG activation [65–67], our functional data for LEP, CD36, and PLIN1 overexpression in breast cancer cells are novel, providing the first experimental evidence of their ability to modulate tumor cell behavior.

Despite the comprehensive multi-omics and experimental approach used in this study, a few limitations should be noted. First, the findings were primarily based on public datasets and in vitro assays, which may not fully capture the complexity of in vivo tumor biology. Second, although we validated gene and miRNA expression in breast cancer cell lines, the lack of patient-derived xenografts or clinical tissue validation limits translational applicability. Third, the functional assays focused only on overexpression models; knockdown or CRISPR-based gene editing could provide more mechanistic insight. Finally, the study does not account for potential subtype-specific effects in triple-negative or HER2-enriched breast cancers, which may respond differently to the hub gene alterations.

## Conclusion

This study provides a comprehensive evaluation of PPARG, LEP, CD36, and PLIN1 through an integrated analysis combining multi-cohort transcriptomic profiling, promoter methylation assessment, pathway activity analysis, genomic alteration data, and in vitro functional validation. All four genes were consistently downregulated in breast cancer across datasets and experimental models, and their reduced expression was associated with poorer patient outcomes. Functional assays further demonstrated that restoring the expression of these genes suppresses proliferation, colony formation, and migration in breast cancer cells, supporting their roles as tumour-suppressive regulators. Together, these findings clarify the coordinated involvement of PPARG, LEP, CD36, and PLIN1 in breast cancer progression and highlight their potential utility as biomarkers and targets for future therapeutic exploration.

## Supplementary Information

Supplementary Material 1.

## Data Availability

Any type of the data will be provided by the corresponding author.
