# Supplementary figures and images for: A combined transcriptomic, epigenetic, and functional analysis identifies novel biomarkers in breast cancer
Source: Hereditas. 2025 Dec 27;163:16. doi: 10.1186/s41065-025-00630-1 (PMC12853850; doi:10.1186/s41065-025-00630-1)

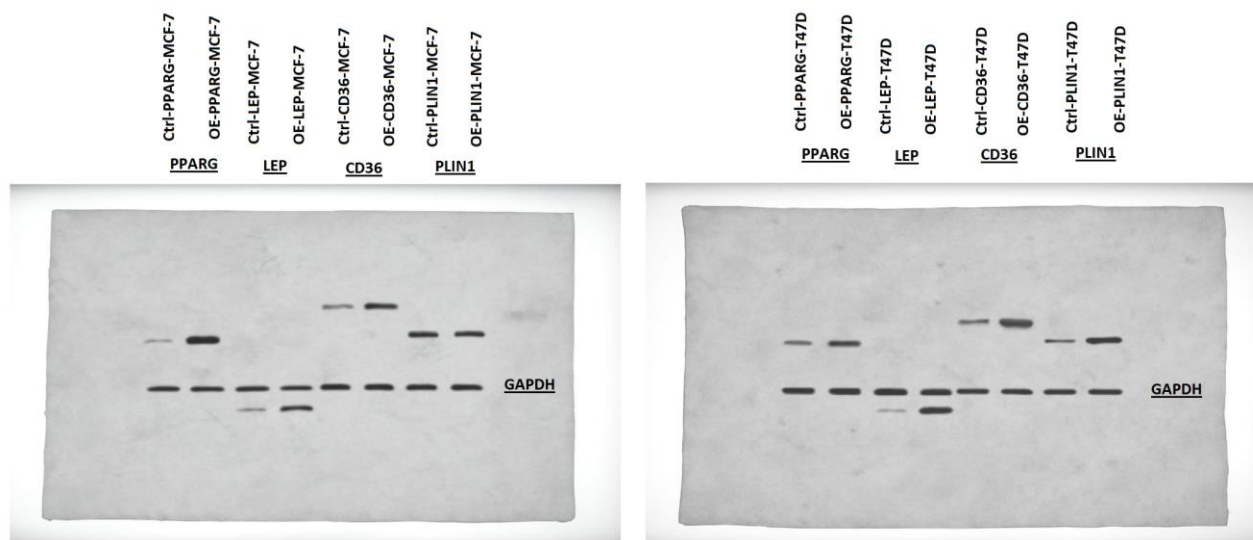

**Supplementary data Figure 1: Uncut Western blot bands of PPARG, LEP, CD36, PLIN1, and GAPDH.**

Supplement: Supplementary file 1 — Supplementary Material 1. [file 41065_2025_630_MOESM1_ESM.pdf]
